# Immunomodulatory Properties of Bacterium-Like Particles Obtained From Immunobiotic Lactobacilli: Prospects for Their Use as Mucosal Adjuvants

**DOI:** 10.3389/fimmu.2020.00015

**Published:** 2020-01-23

**Authors:** Fernanda Raya Tonetti, Lorena Arce, Susana Salva, Susana Alvarez, Hideki Takahashi, Haruki Kitazawa, Maria Guadalupe Vizoso-Pinto, Julio Villena

**Affiliations:** ^1^Infection Biology Lab, Instituto Superior de Investigaciones Biológicas (INSIBIO), CONICET-UNT, Tucumán, Argentina; ^2^Facultad de Medicina, Universidad Nacional de Tucumán (UNT), Tucumán, Argentina; ^3^Laboratorio de Ciencias Básicas & Or. Genética, Facultad de Medicina, Universidad Nacional de Tucumán, Tucumán, Argentina; ^4^Laboratory of Immunobiotechnology, Reference Centre for Lactobacilli (CERELA-CONICET), Tucumán, Argentina; ^5^Laboratory of Plant Pathology, Graduate School of Agricultural Science, Tohoku University, Sendai, Japan; ^6^Plant Immunology Unit, International Education and Research Center for Food Agricultural Immunology, Graduate School of Agricultural Science, Tohoku University, Sendai, Japan; ^7^Food and Feed Immunology Group, Laboratory of Animal Products Chemistry, Graduate School of Agricultural Science, Tohoku University, Sendai, Japan; ^8^Livestock Immunology Unit, International Education and Research Center for Food Agricultural Immunology (CFAI), Graduate School of Agricultural Science, Tohoku University, Sendai, Japan

**Keywords:** lactobacilli, bacterium-like particles, mucosal vaccine, adjuvant, rotavirus

## Abstract

Non-viable lactic acid bacteria (LAB) have been proposed as antigen delivery platforms called bacterium-like particles (BLPs). Most studies have been performed with *Lactococcus lactis*-derived BLPs where multiple antigens were attached to the peptidoglycan surface and used to successfully induce specific immune responses. It is well-established that the immunomodulatory properties of LAB are strain dependent and therefore, the BLPs derived from each individual strain could have different adjuvant capacities. In this work, we obtained BLPs from immunomodulatory (immunobiotics) and non-immunomodulatory *Lactobacillus rhamnosus* and *Lactobacillus plantarum* strains and comparatively evaluated their ability to improve the intestinal and systemic immune responses elicited by an attenuated rotavirus vaccine. Results demonstrated that orally administered BLPs from non-immunomodulatory strains did not induce significant changes in the immune response triggered by rotavirus vaccine in mice. On the contrary, BLPs derived from immunobiotic lactobacilli were able to improve the levels of anti-rotavirus intestinal IgA and serum IgG, the numbers of CD24^+^B220^+^ B and CD4^+^ T cells in Peyer's patches and spleen as well as the production of IFN-γ by immune cells. Interestingly, among immunobiotics-derived BLPs, those obtained from *L. rhamnosus* CRL1505 and *L. rhamnosus* IBL027 enhanced more efficiently the intestinal and systemic humoral immune responses when compared to BLPs from other immunobiotic bacteria. The findings of this work indicate that it is necessary to perform an appropriate selection of BLPs in order to find those with the most efficient adjuvant properties. We propose the term Immunobiotic-like particles (IBLPs) for the BLPs derived from CRL1505 and IBL027 strains that are an excellent alternative for the development of mucosal vaccines.

## Introduction

Lactic acid bacteria (LAB) have been studied for several years as potential delivery systems and/or adjuvants for mucosal vaccine development. In this regard, recombinant LAB expressing pathogen's antigens in their cell-walls have been used as oral or nasal vaccines in animal models (1–4). The mucosal administration of recombinant LAB was shown to elicit specific systemic and mucosal immune responses against selected antigens. However, the administration of genetically modified organisms is associated with ethical concerns related to the possible dissemination of virulence factors or antibiotic resistant genes. Then, similar to other genetically modified organisms, recombinant LAB expressing antigens from pathogens and carrying antibiotic resistant genes cannot be applied in humans.

An original alternative to the use of genetically modified LAB was the development of bacterium-like particles (BLPs) derived from the acid and heat treatments of *Lactococcus lactis* (5–8). These *L. lactis*-derived BLPs, also known as Gram-positive enhancer matrix particles, do not contain DNA or cytoplasmic proteins but conserve the adjuvant capacities of the bacteria. The acid/heat treatment results in the bacterial death, which implies greater safety related to the maximum dose administered, and in addition to causing the loss of genetic material, exposes the peptidoglycan of the cell-wall increasing its capacity to bind proteins with lysine motifs (LysM) at least 10 times (5). The ability of BLPs to improve the protective immunity generated at the mucosal level by recombinant proteins from different pathogens fused to LysM has been widely evaluated. The microbial pathogens tested in those investigations included bacteria, parasites and viruses such as *Streptococcus pneumoniae* (5), *Plasmodium berghei* (7, 9), and Influenza virus (10). Those studies clearly demonstrated that *L. lactis*-derived BLPs are a promising adjuvant for mucosal immunization.

The immunomodulatory properties of LAB proved to depend on each individual strain. Previously, we evaluated the ability of immunomodulatory viable LAB strains, administered by the oral route in the same dose and during the same period, on intestinal immunity (11–13). We found that although both *Lactobacillus rhamnosus* CRL1505 and *Lactobacillus plantarum* CRL1506 improved the intestinal immunity and the protection against pathogens, the CRL1505 strain was more efficient than the CRL1506 to achieve those beneficial effects (11–13). Similarly, both *L. lactis* NZ9000 and *L. rhamnosus* CRL1505 administered by the nasal route were able to improve respiratory immunity and confer protection against *S. pneumoniae* infection. However, the protective effect induced by *L. lactis* NZ9000 was modest when compared to *L. rhamnosus* CRL1505 and it was necessary a longer administration period of lactococci than lactobacilli (14–16).

We hypothesized that BLPs obtained from different immunomodulatory LAB would have different adjuvant capacity when used in mucosal vaccine formulations. In this work, we obtained BLPs from immunomodulatory (immunobiotics), and non-immunomodulatory *L. rhamnosus* and *L. plantarum* strains and comparatively evaluated their ability to improve intestinal and systemic immune responses to an oral attenuated rotavirus vaccine.

## Materials and Methods

### Microorganisms and Bacterium-Like Particles

*L. rhamnosus* CRL1505, *L. plantarum* CRL1506 and *L. plantarum* CRL1905 were obtained from the culture collection of CERELA (Tucumán, Argentina). *L. rhamnosus* IBL027 was obtained from the culture collection of the Infection Biology Laboratory of INSIBIO (Tucumán, Argentina). Lactobacilli (10^10^ CFU stored at −70°C) were activated and cultured for 12 h at 37°C (final log phase) in Man-Rogosa-Sharpe (MRS) broth culture media. The bacteria were harvested by centrifugation and washed with sterile PBS (0.01 mol/L, pH 7.2). Chemical pre-treatment of lactobacilli to generate BLP and immunomodulatory bacterium-like particles (IBLP) was performed as follows. Bacteria from a fresh overnight culture (100 ml) were collected by centrifugation (10 min, 13,000 x g) and washed once with sterile distilled water. Afterwards, the pellet was suspended in 20 ml of 0.1 M HCl and boiled in a water bath for 45 min. Next, the cells were washed three times in 50 ml sterile phosphate buffer saline (PBS), pH 7.4, with vigorous vortexing. After the last washing step, cells were resuspended in 10 ml PBS and stored at −20°C. The number of IBLP particles per milliliter was adjusted according to the CFU/ml determined in the starting culture. Viability of BLPs and IBLPs was checked by plating the suspensions and several dilutions on to MRS agar plates, which were incubated overnight at 37°C in microaerophilia.

### Electron Microscopy

For transmission electron microscopy, samples were prepared according to the Centro de Investigaciones y Servicios de Microscopía Electronica (CISME–CONICET) standard procedure. Briefly, *L. rhamnosus* CRL1505 and IBLP1505 were fixed by adding Karnovsky fixative. After 24 h of fixation at 4°C, samples were washed three times with 0.1M sodium phosphate buffer (pH 7.4) and postfixed overnight in a solution containing 1% osmium tetroxide in sodium phosphate buffer. After dehydratation with a graded ethanol series, the samples were embedded in Spurr resin. Ultrathin sections were cut with an ultramicrotome and examined with a Zeiss Libra 120 transmission electron microscope.

### Animals and Ethical Statement

Four-week-old female BALB/c mice were obtained from the closed colony at CERELA (Tucumán, Argentina) in SPF conditions. Animals were housed in plastic cages and environmental conditions were kept constant, in agreement with the standards for animal housing. Animal welfare was in charge of researchers and special staff trained in animal care and handling at CERELA. Animal health and behavior were monitored twice a day.

This study was carried out in strict accordance with the recommendations in the Guide for the Care and Use of Laboratory Animals of the Guidelines for Animal Experimentation of CERELA. The CERELA Institutional Animal Care and Use Committee prospectively approved this research under the protocol BIOT-CRL-18.

### Immunization Protocols

Mice were vaccinated with the commercial, oral, pentavalent, and live rotavirus vaccine RotaTeq® (Merck & Co., Inc.). Two sets of experiments with different immunization protocols were used. In the first set of experiments, mice were vaccinated with 30 μl of rotavirus vaccine and 10^8^ bacterial cells or particles (high vaccine/low adjuvant immunization protocol). Mice that received only 30 μl of rotavirus vaccine were used as controls. In a second set of experiments, mice were vaccinated with 7.5 μl of rotavirus vaccine and 10^9^ bacterial cells or particles (low vaccine/high adjuvant immunization protocol). Mice that received only 7.5 μl of rotavirus vaccine were used as controls. In both protocols, mice were immunized orally on days 0, 14 and 28. Seven days after the last immunization (day 35), the specific humoral and cellular immune responses were evaluated as described below.

### Serum and Intestinal Antibodies

Blood samples were obtained through cardiac puncture seven days after the last immunization (day 35) and collected in heparinized tubes. Intestinal fluid samples were obtained as described previously (11). Briefly, the small intestine was flushed with 5 ml of PBS and the fluid was centrifuged (10,000×g 4°C for 10 min) to separate particulate material. The supernatant was kept frozen until use. Specific anti-rotavirus antibodies (IgA and IgG) were determined by ELISA. Plates were coated with 1.5 μg of Rotateq per well overnight at 4°C and blocked with serum bovine albumin. Appropriate dilutions of the samples (serum 1:20; intestinal fluid 1:2) were incubated for 1 h at 37°C. Peroxidase conjugated anti-mouse IgG, or IgA antibodies (1:500) (Sigma-Aldrich) were added and incubated for 1 h at 37°C. The reaction was developed with TMB Substrate Reagent (Sigma-Aldrich) and measured at 450 nm in a microplate reader.

### Flow Cytometry

Ilium Payer's patches and spleens were collected and mechanically disaggregated. A single-cell suspension from the Peyer's patches or spleens of each mouse was obtained by gently passing the collected tissue through a tissue strainer with PBS supplemented with 2% FCS (FACS buffer). Cell suspensions were subjected to red blood cells lysis (Tris-ammonium chloride, BD PharMingen) and counted on a hemocytometer. Trypan blue exclusion method was used to assess viability of cells. Cell suspensions were pre-incubated with anti-mouse CD32/CD16 monoclonal antibody (Fc block) for 30 min at 4°C. Cells were incubated with the antibody mixes for 30 min at 4°C and washed with FACS buffer. The following antibodies from BD Biosciences were used: PE-labeled anti-mouse CD24, biotinylated anti-mouse B220, PE-labeled anti-mouse CD45, biotinylated anti-mouse CD45, FITC-labeled anti-mouse CD3, PE-labeled anti-mouse CD8, and biotinylated anti-mouse CD4 antibodies. Streptavidin-PerCP was used as a second-step reagent. Flow cytometry was performed using a BD FACSCaliburTM flow cytometer (BD Biosciences) and data were analyzed using FlowJo software (TreeStar).

### Cytokine Production by Immune Cells

Ilium Payer's patches and spleens were collected, and a single-cell suspension from each mouse was obtained as described above. After red blood cells lysis, isolated cells were suspended in complete DMEM (Invitrogen) supplemented with 10% FCS (Sigma), 50 μg/ml penicillin-streptomycin, and 50 μg/ml gentamicin (Invitrogen). Cells (4 × 10^6^ cells/well) were cultured in 24-well plates in the presence of 0.5 μl of the rotavirus vaccine. Cytokines were quantified in culture supernatants by ELISA after 24 h at 37°C. The concentrations of tumor necrosis factor (TNF)-α, interferon (IFN)-γ, and interleukin (IL)-4 were measured on the supernatants of the stimulated mononuclear cells isolated from Peyer's patches or spleen with commercially available enzyme-linked immunosorbent assay (ELISA) kits following the manufacturer's recommendations (R&D Systems, MN, USA).

### Statistical Analysis

Each experimental group consisted of 3 mice per group at each time point and experiments were performed in triplicate (*n* = 9 for each parameter studied). Results were expressed as mean ± standard deviation (SD). The differences between groups were analyzed using student *t*-test. Differences were considered significant at *p* < 0.05. ANOVA one-way was used for analysis of variance among multiple groups.

## Results

### Development of IBLP From *L. rhamnosus* CRL1505

**Figure 1:**
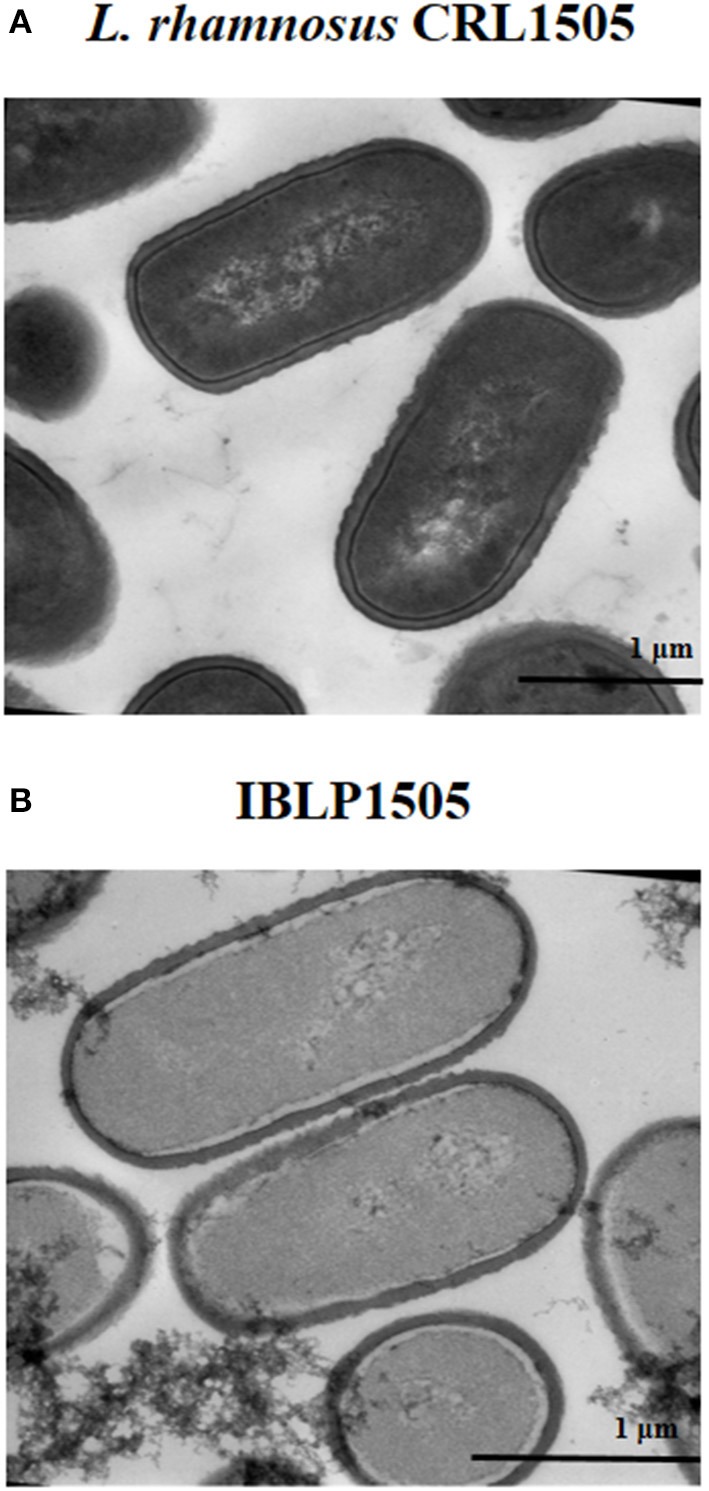
Transmission electron microscopy analysis. **(A)** *Lactobacillus rhamnosus* CRL1505 untreated cells and **(B)** bacterium-like particles obtained from *L. rhamnosus* CRL1505 (IBLP1505) were fixed with Karnovsky fixative, postfixed with 1% osmium tetroxide in sodium phosphate buffer and embedded in Spurr resin. Ultrathin sections cuts were examined with a Zeiss libra 120 Transmission Electron Microscope.

The heat-acid treatment of the lactobacilli results in non-living particles. This procedure most likely affected the protein and DNA content of the IBLPs. The intracellular contents of the IBLPs seemed to be partially released or degraded (Figure 1). IBLPs have the same size and shape than living lactobacilli. Peptidoglycan, the main component of the lactobacilli's cell wall, was exposed by the harsh acid treatment. This peptidoglycan matrix preserved the structural integrity of lactobacilli (Figure 1).

### IBLP From *L. rhamnosus* CRL1505 Enhance the Humoral Immune Response Against Rotavirus Vaccine

**Figure 2:**
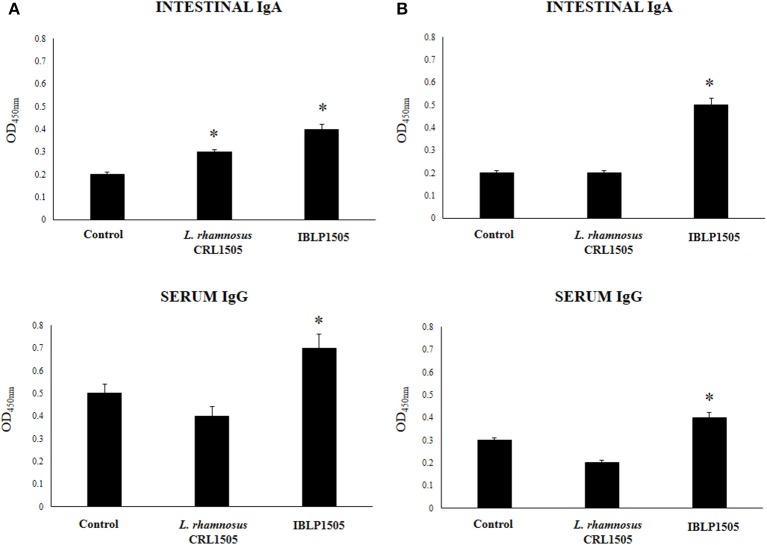
Effect of immunobiotic bacterium-like particles obtained from *Lactobacillus rhamnosus* CRL1505 (IBLP1505) on the humoral immune response induced by the immunization of mice with an oral rotavirus vaccine. Mice (4-week-old) were immunized on days 0, 14, and 28 by the oral route with the rotavirus vaccine and IBLP1505 as adjuvant. Mice orally vaccinated with rotavirus vaccine only (controls) and mice receiving the vaccine with viable *L. rhamnosus* CRL1505 as adjuvant were used for comparisons. Two immunization protocols were used: **(A)** each mouse was vaccinated with 30 μl of vaccine and 10^8^ bacterial cells or particles; **(B)** each mouse was vaccinated with 7.5 μl of vaccine and 10^9^ bacterial cells or particles. Seven days after the last immunization, serum, and intestinal fluid samples were obtained for the determination of IgA and IgG specific antibodies. Each experimental group consisted of 3 mice per group and experiments were performed in triplicate (*n* = 9). Results were expressed as mean ± standard deviation. Differences were considered significant at *P* < 0.05 when compared with animals immunized with rotavirus vaccine only (*).

In order to evaluate the adjuvant capacity of IBLP1505, we selected an oral pentavalent vaccine that contains five live reassortant rotaviruses. Then, mice were immunized on days 0, 14, and 28 by the oral route with the rotavirus vaccine and IBLP1505 as described in detail in the materials and methods section. Mice orally immunized with the vaccine alone or added with viable *L. rhamnosus* CRL1505 were used for comparisons. The levels of rotavirus-specific intestinal IgA and serum IgG were determined seven days after the last immunization. In a first set of experiments, mice were vaccinated with 30 μl of vaccine and 10^8^ bacterial cells or particles (high vaccine/low adjuvant immunization protocol). As shown in Figure 2A, both intestinal and serum specific antibodies were detected in control mice indicating that the vaccine induced mucosal and systemic humoral immune responses in our model. The levels of rotavirus-specific intestinal IgA and serum IgG in mice immunized with the rotavirus vaccine and IBLP1505 were significantly higher when compared to controls (Figure 2A). In addition, intestinal IgA in mice immunized with the rotavirus vaccine and viable *L. rhamnosus* CRL1505 were significantly higher than controls. However, in this group of mice, the levels of serum specific IgG antibodies were not different from controls (Figure 2A).

In a second set of experiments, we evaluated whether the dose of rotavirus vaccine could be reduced by using a higher dose of IBLP1505 (low vaccine/high adjuvant immunization protocol). Then, mice were vaccinated with 7.5 μl of vaccine and 10^9^ bacterial cells or particles. As shown in Figure 2B, the levels of intestinal rotavirus-specific IgA did not change in comparison with the previous vaccination protocol. However, a significant reduction of serum specific IgG antibodies was detected. The levels of intestinal IgA and serum IgG antibodies in mice immunized with the rotavirus vaccine and IBLP1505 were significantly higher than controls (Figure 2B). We also observed that intestinal IgA levels were higher and that serum IgG levels were lower than the values obtained in the first immunization protocol (Figure 2A). Interestingly, the group of mice receiving the low vaccine dose together with viable *L. rhamnosus* CRL1505 had rotavirus-specific intestinal IgA and serum IgG antibodies that were not different from controls (Figure 2B).

### IBLP From *L. rhamnosus* CRL1505 Enhance the Mucosal Cellular Immune Response Against Rotavirus Vaccine

**Figure 3:**
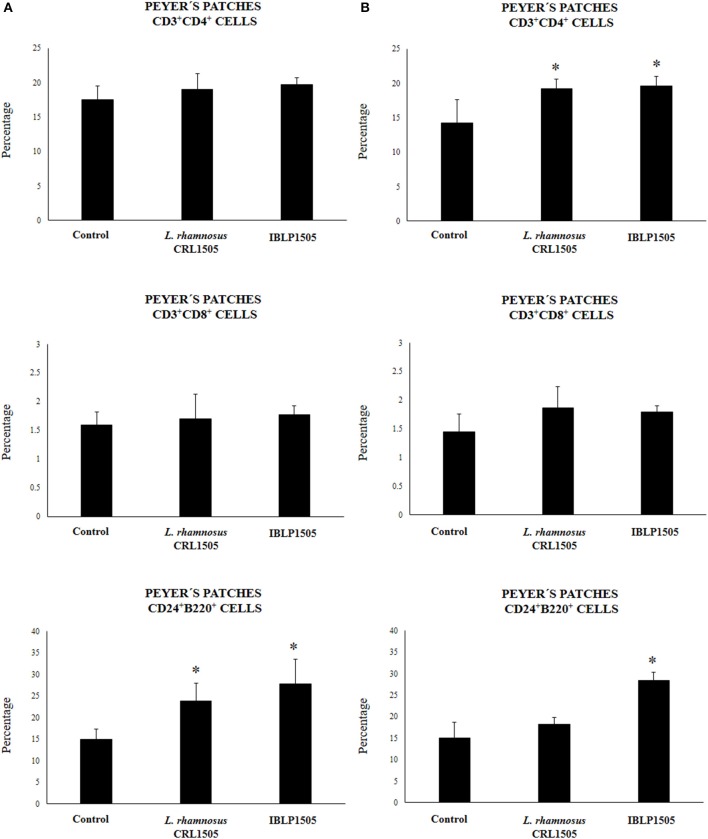
Effect of immunobiotic bacterium-like particles obtained from *Lactobacillus rhamnosus* CRL1505 (IBLP1505) on the cellular immune response induced by the immunization of mice with an oral rotavirus vaccine. Mice (4-week-old) were immunized on days 0, 14, and 28 by the oral route with the rotavirus vaccine and IBLP1505 as adjuvant. Mice orally vaccinated with rotavirus vaccine only (controls) and mice receiving the vaccine with viable *L. rhamnosus* CRL1505 as adjuvant were used for comparisons. Two immunization protocols were used: **(A)** each mouse was vaccinated with 30 μl of vaccine and 10^8^ bacterial cells or particles; **(B)** each mouse was vaccinated with 7.5 μl of vaccine and 10^9^ bacterial cells or particles. Seven days after the last immunization, Peyer's patches samples were obtained for the study of CD3^+^CD4^+^, CD3^+^CD8^+^ T and CD24^+^B220^+^ B cells within the CD45^+^ population by flow cytometry. Each experimental group consisted of 3 mice per group and experiments were performed in triplicate (*n* = 9). Results were expressed as mean ± standard deviation. Differences were considered significant at *P* < 0.05 when compared with animals immunized with rotavirus vaccine only (*).

We next evaluated whether the two immunization protocols were able to modify the mucosal and systemic cellular immune elicited by rotavirus vaccine. First, we evaluated the proportion of CD3^+^CD4^+^ and CD3^+^CD8^+^ T cells as well as CD24^+^B220^+^ B cells in Peyer's patches of vaccinated mice. In the high vaccine/low IBLP1505 immunization protocol (Figure 3A), the vaccination of control mice did not induce significant changes in CD3^+^CD4^+^, CD3^+^CD8^+^, or CD24^+^B220^+^ cells percentages when compared with non-immunized mice (data not shown). In addition, no differences were observed in CD3^+^CD4^+^ and CD3^+^CD8^+^ T cells when mice immunized with rotavirus vaccine and IBLP1505 or viable *L. rhamnosus* CRL1505 were compared to controls. However, the vaccination protocols that included IBLP1505 or viable *L. rhamnosus* CRL1505 as adjuvants significantly increased the proportion of CD24^+^B220^+^ B cells in Peyer's patches when compared to controls (Figure 3A). In the low vaccine/high adjuvant immunization protocol (Figure 3B), the vaccination of mice with the rotavirus vaccine and IBLP1505 significantly increased CD3^+^CD4^+^ T cells as well as CD24^+^B220^+^ B cells in Peyer's patches. Mice, which received rotavirus vaccine and viable *L. rhamnosus* CRL1505 only had significantly higher CD3^+^CD4^+^ T cells than controls (Figure 3B). No significant differences between the groups were observed regarding CD3^+^CD8^+^ T cell counts.

**Figure 4:**
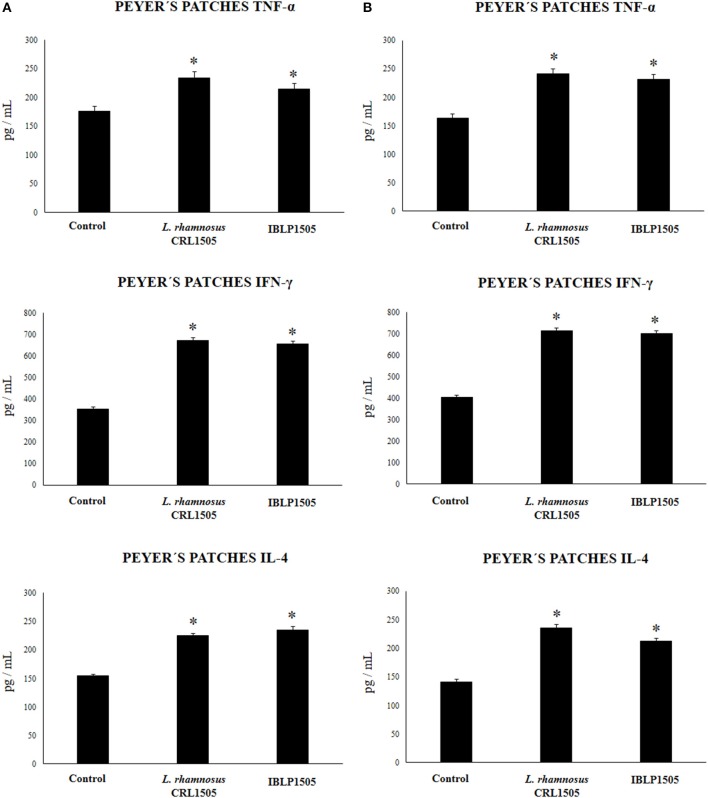
Effect of immunobiotic bacterium-like particles obtained from *Lactobacillus rhamnosus* CRL1505 (IBLP1505) on the cellular immune response induced by the immunization of mice with an oral rotavirus vaccine. Mice (4-week-old) were immunized on days 0, 14, and 28 by the oral route with the rotavirus vaccine and IBLP1505 as adjuvant. Mice orally vaccinated with rotavirus vaccine only (controls) and mice receiving the vaccine with viable *L. rhamnosus* CRL1505 as adjuvant were used for comparisons. Two immunization protocols were used: **(A)** each mouse was vaccinated with 30 μl of vaccine and 10^8^ bacterial cells or particles; **(B)** each mouse was vaccinated with 7.5 μl of vaccine and 10^9^ bacterial cells or particles. Seven days after the last immunization, immune cells from Peyer's patches were isolated and *in vitro* stimulated with rotavirus vaccine. Tumor necrosis factor (TNF)-α, interferon (IFN)-γ and interleukin (IL)-4 were measured in culture supernatants. Each experimental group consisted of 3 mice per group and experiments were performed in triplicate (*n* = 9). Results were expressed as mean ± standard deviation. Differences were considered significant at *P* < 0.05 when compared with animals immunized with rotavirus vaccine only (*).

We then assessed the ability of immune cells obtained from the Peyer's patches of vaccinated mice to induce cytokines in response to the *ex vivo* stimulation with the rotavirus vaccine. For this purpose, isolated immune cells were cultured and after challenging them with rotavirus the levels of TNF-α, IFN-γ and IL-4 were evaluated in the supernatants (Figure 4). As expected, the vaccination of control mice with the two protocols used in this work significantly increased the production of TNF-α, IFN-γ, and IL-4 by Peyer's patches immune cells in response to vaccine stimulation when compared with non-immunized mice (data not shown). In addition, both the high vaccine/low IBLP1505 and the low vaccine/high IBLP1505 immunization protocols significantly enhanced the production of TNF-α, IFN-γ, and IL-4 when compared to the control group (Figure 4). Similarly, the vaccination protocols using viable *L. rhamnosus* CRL1505 as adjuvant increased the levels of these cytokines when compared to control mice (Figure 4).

### IBLP From *L. rhamnosus* CRL1505 Enhance the Systemic Cellular Immune Response Against Rotavirus Vaccine

**Figure 5:**
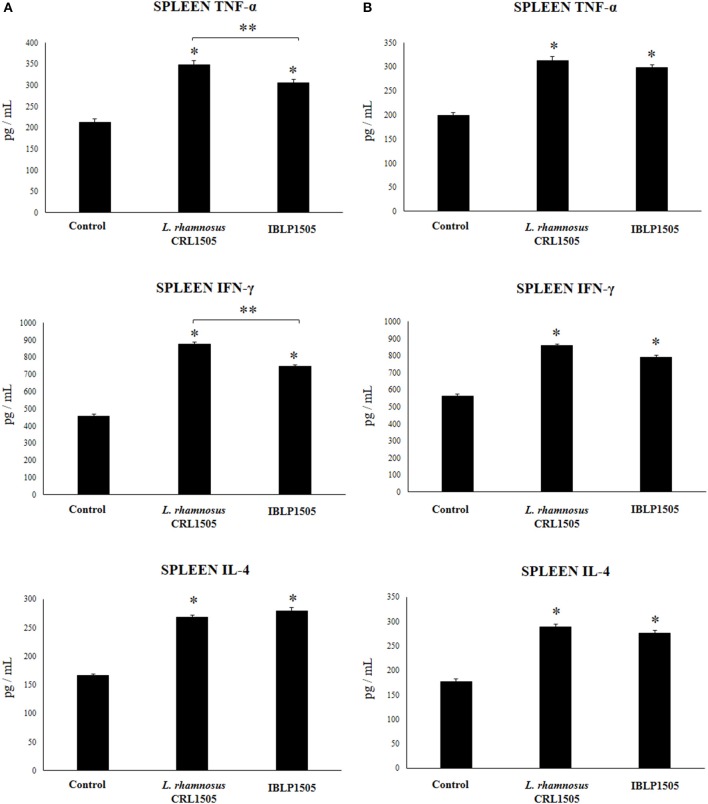
Effect of immunobiotic bacterium-like particles obtained from *Lactobacillus rhamnosus* CRL1505 (IBLP1505) on the cellular immune response induced by the immunization of mice with an oral rotavirus vaccine. Mice (4-week-old) were immunized on days 0, 14, and 28 by the oral route with the rotavirus vaccine and IBLP1505 as adjuvant. Mice orally vaccinated with rotavirus vaccine only (controls) and mice receiving the vaccine with viable *L. rhamnosus* CRL1505 as adjuvant were used for comparisons. Two immunization protocols were used: **(A)** each mouse was vaccinated with 30 μl of vaccine and 10^8^ bacterial cells or particles; **(B)** each mouse was vaccinated with 7.5 μl of vaccine and 10^9^ bacterial cells or particles. Seven days after the last immunization, immune cells from spleens were isolated and *in vitro* stimulated with rotavirus vaccine. Tumor necrosis factor (TNF)-α, interferon (IFN)-γ and interleukin (IL)-4 were measured in culture supernatants. Each experimental group consisted of 3 mice per group and experiments were performed in triplicate (*n* = 9). Results were expressed as mean ± standard deviation. Differences were considered significant at *P* < 0.05 when compared with animals immunized with rotavirus vaccine only (*) or between the indicated groups (**).

The proportions of CD3^+^CD4^+^ and CD3^+^CD8^+^ T cells as well as CD24^+^B220^+^ B cells in spleen of vaccinated mice were also evaluated. No significant differences were observed between the groups when these immune cell populations were evaluated for both immunization protocols (data not shown). In addition, the levels of TNF-α, IFN-γ, and IL-4 were quantified in the supernatants of cultured isolated immune cells from spleens after the challenge with the rotavirus vaccine (Figure 5). In the high vaccine/low IBLP1505 or viable *L. rhamnosus* CRL1505 immunization protocols (Figure 5A), the vaccination with rotavirus vaccine and adjuvants significantly increased the production of TNF-α, IFN-γ, and IL-4 by splenocytes when compared to controls. Interestingly, the levels of TNF-α and IFN-γ were significantly higher in mice immunized with viable *L. rhamnosus* CRL1505 than in those receiving IBLP1505 (Figure 5A). On the other hand, in the low vaccine/high IBLP1505 or viable *L. rhamnosus* CRL1505 immunization protocols (Figure 5B), the vaccination of mice significantly enhanced the production of the three cytokines by splenocytes when compared to controls.

### The Adjuvant Capacities of BLP From Lactobacilli Are Strain-Dependent

**Figure 6:**
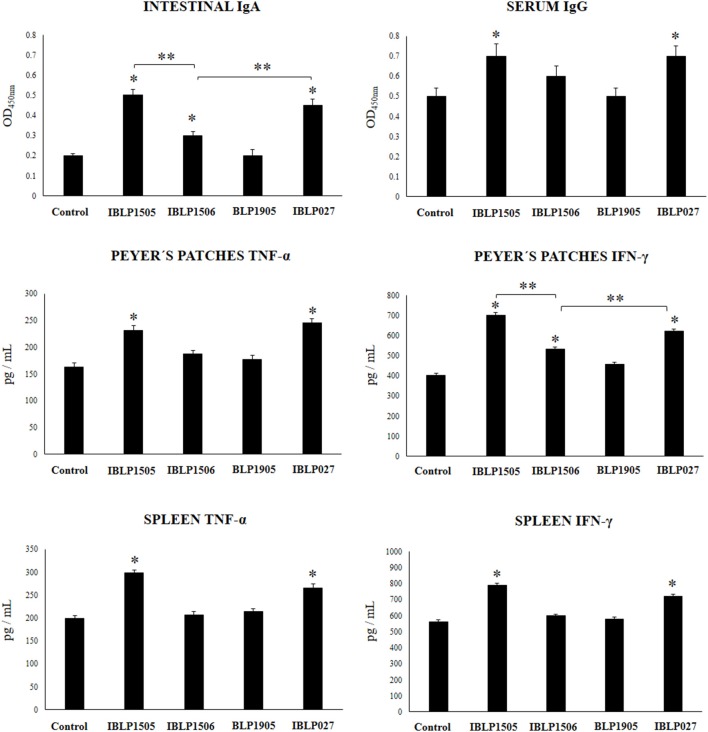
Effect of bacterium-like particles obtained from different lactobacilli strains on the immune response triggered by the immunization of mice with an oral rotavirus vaccine. Mice (4-week-old) were immunized on days 0, 14, and 28 by the oral route with the rotavirus vaccine and bacterium-like particles from obtained from the immunomodulatory strains *L. rhamnosus* CRL1505 (IBLP1505), *L. rhamnosus* IBL027 (IBLP027), and *L. plantarum* CRL1506 (IBLP1506) and from the non-immunomodulatory strain *L. plantarum* CRL1905 (IBLP1905) as adjuvants. Mice orally vaccinated with rotavirus vaccine only (controls) were used for comparisons. Each mouse was vaccinated with 7.5 μl of vaccine and 10^9^ bacterial particles. Seven days after the last immunization, serum, and intestinal fluid samples were obtained for the determination of IgA and IgG specific antibodies. In addition, immune cells from Peyer's patches and spleen were isolated, cultured and stimulated with rotaviral vaccine. TNF-α, and IFN-γ concentrations were determined in the supernatants by ELISA. Each experimental group consisted of 3 mice per group and experiments were performed in triplicate (*n* = 9). Results were expressed as mean ± standard deviation. Differences were considered significant at *P* < 0.05 when compared with animals immunized with rotavirus vaccine only (*) or between the indicated groups (**).

Finally, we aimed to evaluate whether the adjuvant capacity of IBLP1505 was a strain characteristic or if it was shared by BLP derived from other immunomodulatory strains. Then, we obtained BLP from the immunomodulatory strains *L. rhamnosus* IBL027 and *L. plantarum* CRL1506 and from the non-immunomodulatory strain *L. plantarum* CRL1905. The particles were designated as IBLP027, IBLP1506, and BLP1905 and used to immunize mice with the low vaccine/high adjuvant immunization protocol. As shown in Figure 6, IBLP027 was as effective as IBLP1505 to improve the production of rotavirus-specific intestinal IgA and serum IgG antibodies. In addition, intestinal IgA in mice immunized with the rotavirus vaccine and IBLP1506 were significantly higher than controls. However, these antibodies were lower when compared to those in mice receiving IBLP027 or IBLP1505 (Figure 6). The immunization with BLP1905 was not able to significantly change the levels of rotavirus-specific intestinal IgA and serum IgG antibodies when compared to controls.

In addition, we assessed the ability of immune cells obtained from the Peyer's patches and spleens of mice vaccinated with IBLP027, IBLP1506, or BLP1905 to induce TNF-α, IFN-γ and IL-4 in response to the *ex vivo* stimulation with rotavirus (Figure 6). IBLP027 was as effective as IBLP1505 to improve the production of TNF-α and IFN-γ by intestinal and spleen immune cells in response to rotavirus challenge. Vaccination with the rotavirus vaccine and IBLP1506 increased the levels of IFN-γ produced by Peyer's patches cells. However, this cytokine was significantly lower when compared to the observed in mice receiving IBLP027 or IBLP1505 (Figure 6). The immunization with BLP1905 was not able to significantly change the levels of TNF-α and IFN-γ produced by intestinal or spleen cells when compared to controls. Similar to IBLP1505 (Figures 4, 5), IBLP027, IBLP1506, and BLP1905 improved the levels of IL-4 in immune cells obtained from the Payer's patches and spleens. There was no statistical differences among the groups (data not shown).

## Discussion

There is an urgent need to develop effective vaccines to reduce the global burden of infectious disease in both humans and animals. The use of mucosal vaccines triggers local and systemic responses limiting the pathogens at the site of entry, reducing their replication in epithelial cells, the alteration of the epithelial barrier, and the action of toxins on the mucosa. In addition, mucosal vaccination helps preventing the spread of the pathogen to internal tissues and to the environment (17, 18). Despite the several advantages of mucosal vaccination, it faces many difficulties including poor immunogenicity at low concentrations, chemical and enzymatic degradation, potential toxicity, and the risk of generating tolerance rather than protective immunity (17, 19). In this work, we demonstrated that IBLP derived from highly efficient immunomodulatory lactobacilli are an interesting alternative as mucosal adjuvants.

It is well-documented that within the gastrointestinal tract, immunomodulatory LAB are capable to interact with the host's epithelial and immune cells, and thereby beneficially influence epithelial barrier and immune functions (20). It has been proposed that the final outcome of a host cell response against beneficial immunomodulatory microorganisms depends on the combination of distinct microbial-associated molecular patterns (MAMPs) that can interact with various pattern recognition receptors (PRRs) and associated co-receptors to trigger different signaling pathways (20, 21). The unique combination of cellular and molecular interactions that are established between a certain microorganism with the host cells explains why the immunomodulatory properties of LAB are a strain dependent characteristic. The results of the present work strongly suggest that the same principle could be applied to IBLP derived from different LAB strains. In our hands, IBLP derived from immunomodulatory *L. rhamnosus* CRL1505, *L. rhamnosus* IBL027 and *L. plantarum* CRL1506 showed adjuvant capacities when used together with the rotavirus vaccine while the particles derived from the non-immunomodulatory *L. plantarum* CRL1905 strain were not able to induce modifications in the immune response triggered by the oral vaccination. Moreover, our experiments demonstrated that the particles derived from the immunomodulatory *Lactobacillus* strains had different adjuvant capacities in terms of their ability to improve the cellular and humoral immune responses triggered by rotavirus vaccination.

Our previous comparative *in vitro* studies evaluating the ability of *L. rhamnosus* CRL1505 and *L. plantarum* CRL1506 to modulate immune responses in intestinal epithelial cells and antigen presenting cells (APCs) found significant differences between the two lactobacilli strains (22, 23). It was demonstrated that intestinal epithelial cells were modulated by immunobiotic CRL1505 and CRL1506 in a strain-dependent fashion to enhance antiviral responses (23). Interestingly, *L. rhamnosus* CRL1505 was more efficient that *L. plantarum* CRL1506 to increase the expression of IFN-β and IL-6 in intestinal epithelial cells, both cytokines known to influence immune responses generated by immune cells located under the epithelium. In addition, both lactobacilli strains were reported to functionally modulate APCs from porcine Peyer's patches. However, *L. rhamnosus* CRL1505 was more efficient than *L. plantarum* CRL1506 to improve the expression of IL-1β, IL-6, and IFN-γ in porcine CD172a^+^CD11R1^high^ and CD172a^−^CD11R1^low^ dendritic cells (22). The improved Th1 response induced by *L. rhamnosus* CRL1505 in porcine APCs was triggered by TLR2 signaling and included augmented expression of MHC-II and co-stimulatory molecules. Then, our previous results would indicate that IBLP1505 is more efficient than IBLP1506 to modulate the response of intestinal epithelial cells and APCs to the challenge with attenuated rotavirus. Moreover, it is tempting to speculate that IBLP1505 and IBLP027 are able to interact more efficiently with intestinal APCs than IBLP1506, inducing an increase in their activation and antigenic presentation capacity, and generating effector immune responses. In fact, both humoral and cellular intestinal specific immune responses were significantly improved in mice immunized with rotavirus vaccine and IBLP1505 or IBLP027 than in animals treated with the vaccine and IBLP1506, indicating the different capacity of the particles to serve as effective mucosal adjuvants.

Recombinant LAB expressing rotavirus antigens have been evaluated as vaccine candidates (24–27). *L. casei* ATCC 393 (24) and *L. lactis* NZ9000 (25) expressing the major protective antigen VP4 from rotavirus efficiently induced the production of serum IgG and mucosal IgA anti-VP4 antibodies, which also demonstrated neutralizing effects on rotavirus infection. Rodríguez-Díaz et al. (26), evaluated the effect of the oral immunization of mice with *L. lactis* expressing the rotavirus VP8 protein intracellularly and extracellularly. Low mucosal IgA synthesis was found only when the secreting *L. lactis* strain was used. On the other hand, Esteban et al. (27), evaluated the immunogenicity of the rotavirus VP6 protein expressed on the surface of *L. lactis*. Authors described the ability of the recombinant *L. lactis* VP6 to significantly increase the levels of specific serum IgG antibodies although it should be mentioned that the researchers used the subcutaneous route for the immunization of mice. Despite the positive results obtained with the aforementioned recombinant bacteria, there is still general concern about the massive use of genetically modified microorganisms because of the possibility of the introduction of exogenous DNA in the intestinal microbiota, especially plasmids that confer antibiotic resistance. The IBLPs evaluated in this work can help to alleviate these concerns associated to recombinant bacteria because they are non-genetically modified and non-living.

By using a directed chromosomal integration approach, Yin et al. (28) were able to develop a stable *L. casei* strain expressing the porcine rotavirus VP4 antigen. The oral immunization of mice with recombinant *L. casei* VP4 induced both specific intestinal and systemic humoral immune responses. Although this improved chromosome recombination and gene expression system could represent an efficient method for safe vaccines production, it would be necessary to design a recombinant bacterium for each vaccine antigen (from the same virus or from different viruses), which would significantly increase the cost of these types of vaccines. On the other hand, IBLPs are able to incorporate efficiently one or more different antigens on their surfaces, which gives them an additional advantage over recombinant bacteria, even with those that could be considered safe for human use.

To the best of our knowledge, few reports have evaluated the ability of non-living LAB as mucosal adjuvants and/or delivery antigens for rotavirus vaccine development. Temprana et al. (29), offered an option to the use of genetically modified organisms in immunizations by obtaining cell wall-derived fragments by mechanical rupture of a recombinant *L. lactis* NZ9000 expressing a cell wall-anchored version of the rotavirus VP6 protein. Authors evaluated the ability of those cell wall-derived fragments containing the VP6 protein to induce specific immunity in mice by using intragrastric immunizations. The report indicated that it was not possible to detect VP6-specific serum IgG or IgA in the mice immunized intragastrically, even when additional mucosal adjuvants were used. In addition, the humoral immune response induced by the experimental vaccine was evaluated while its impact on the specific cellular immune response was not studied. In this work, we offer a different alternative, which can be administered orally and efficiently potentiate the local and systemic immune responses against rotavirus. Furthermore, we demonstrate that our strategy not only stimulate the humoral immunity but in addition, a specific Th1 mucosal response can be also generated against the antigens administered with IBLP. Our results suggest that the adjuvant potential of IBLPs could also be exploited for subunit vaccines, as it was the case of the hepatitis E virus (HEV) capsid protein (ORF2) orally administered with IBLP1505 or IBLP027, which induced both antigen-specific humoral and cellular immune responses in mice (30). Then, the evaluation of the ability of rotavirus antigens co-administered with IBLP or fused to a LysM domain and expressed on the surface of IBLP to generate specific protective immune responses is an interesting topic of on-going research.

Some studies have demonstrated that the administration of viable immunobiotic lactobacilli with attenuated rotavirus vaccines significantly augment the ability of the vaccine to stimulate the specific humoral immune response. In this regard, by using a neonatal gnotobiotic pig model, Wen et al. (31) demonstrated that orally administered *L. rhamnosus* GG increase the levels of virus-specific intestinal IgA after vaccination with attenuated human rotavirus. Similarly, *L. acidophilus* NCFM improved rotavirus-specific antibody production as well as memory B-cell responses to attenuated rotavirus vaccine (32). In line with those studies, we showed here that orally administered viable *L. rhamnosus* CRL1505 improved the humoral immune response of rotavirus-vaccinated mice. Interestingly, mice immunized with rotavirus vaccine and viable *L. rhamnosus* CRL1505 showed lower intestinal IgA and serum IgG specific antibodies than mice vaccinated with IBLP1505 as mucosal adjuvant. This phenomenon could be related to the great ability of viable *L. rhamnosus* CRL1505 to stimulate the innate antiviral intestinal immune responses (11–13, 22). Transcriptomic studies revealed the capacity of *L. rhamnosus* CRL1505 to differentially modulate the innate antiviral immune response in porcine intestinal epithelial cells. Higher expression levels of type I IFNs as well as in several antiviral factors including *ifit1, nlpr3, mda5, msx1, rig1 ifit2*, and *mx2* were found in CRL1505-treated cells when compared to control cells (23). In addition, it was demonstrated *in vitro* that viable *L. rhamnosus* CRL1505 is able to efficiently stimulate the expression of IFN-γ in porcine intestinal CD172a^+^CD11R1^−^ macrophages (22). Then, we hypothesize that the administration of viable *L. rhamnosus* CRL1505 to mice would improve those antiviral mechanisms in the intestinal mucosa, allowing a rapid an efficient elimination of attenuated rotavirus included in the vaccine formulation. The effective elimination of the attenuated pathogens by the innate immune mechanisms would be responsible for the lower interaction of rotavirus with DCs and therefore for the lower stimulation of the adaptive immune response. These results indicate that IBLP1505 has an advantage with respect to viable bacteria to stimulate the intestinal and systemic specific adaptive immune responses to attenuated virus. However, it is important to investigate and compare the ability of viable *L. rhamnosus* CRL1505 and IBLP1505 to stimulate the adaptive immune responses when subunit vaccines are used instead of vaccines based on attenuated pathogens.

There is a growing need for the development of new and improved mucosal vaccines to diminish the morbidity and mortality associated to infectious diseases, particularly against those targeting the respiratory and gastrointestinal tracts (17, 18). The search and characterization of mucosal adjuvants to enhance the immunity against antigens is of fundamental importance to advance in this path. In this regard, BLPs obtained from immunomodulatory beneficial microorganisms are an interesting alternative. Our findings indicate that an appropriate selection of BLPs is necessary in order to find those with the most efficient adjuvant properties. We propose the term Immunobiotic-like particles (IBLPs) for the BLPs derived from highly immunomodulatory strains such as *L. rhamnosus* CRL1505 and *L. rhamnosus* IBL027 that are a promising alternative for the development of mucosal vaccines.

## Data Availability Statement

The datasets generated for this study are available on request to the corresponding author.

## Ethics Statement

The animal study was reviewed and approved by the CERELA Institutional Animal Care and Use Committee under the protocol BIOT-CRL-18. This study was carried out in strict accordance with the recommendations in the Guide for the Care and Use of Laboratory Animals of the Guidelines for Animal Experimentation of CERELA.

## Author's Note

FR and LA hold CONICET scholarships. SS, SA, MV-P, and JV are members of CONICET.

## Author Contributions

JV and MV-P designed the study and wrote the manuscript. FR, LA, and SS did the experiments. MV-P, JV, HK, and HT provided financial support. JV, MV-P, HK, and SA contributed to data analysis and results interpretation.

### Conflict of Interest

The authors declare that the research was conducted in the absence of any commercial or financial relationships that could be construed as a potential conflict of interest.

## References

[1] Bermudez-Humaran LG Cortes-Perez NG Le Loir Y Alcocer-Gonzalez JM Tamez-Guerra RS de Oca-Luna RM. An inducible surface presentation system improves cellular immunity against human papillomavirus type 16 E7 antigen in mice after nasal administration with recombinant lactococci. J Med Microbiol. (2004) 53:427–33. 10.1099/jmm.0.05472-0 15096553

[2] Bermudez-Humaran LG Cortes-Perez NG Le Loir Y Gruss A Rodriguez-Padilla C Saucedo-Cardenas O. Fusion to a carrier protein and a synthetic propeptide enhances E7 HPV-16 production and secretion in *Lactococcus lactis*. Biotechnol Prog. (2003) 19:1101–4. 10.1021/bp0340077 12790689

[3] Villena J Medina M Raya R Alvarez S. Oral immunization with recombinant *Lactococcus lactis* confers protection against respiratory pneumococcal infection. Can J Microbiol. (2008) 54:845–53. 10.1139/W08-077 18923553

[4] Szatraj K Szczepankowska AK Chmielewska-Jeznach M. Lactic acid bacteria - promising vaccine vectors: possibilities, limitations, doubts. J Appl Microbiol. (2017) 123:325–39. 10.1111/jam.13446 28295939 PMC7166332

[5] Audouy SA van Roosmalen ML Neef J Kanninga R Post E van Deemter M. *Lactococcus lactis* GEM particles displaying pneumococcal antigens induce local and systemic immune responses following intranasal immunization. Vaccine. (2006) 24:5434–41. 10.1016/j.vaccine.2006.03.054 16757068

[6] Bosma T Kanninga R Neef J Audouy SA van Roosmalen ML Steen A. Novel surface display system for proteins on non-genetically modified gram-positive bacteria. Appl Environ Microbiol. (2006) 72:880–9. 10.1128/AEM.72.1.880-889.2006 16391130 PMC1352190

[7] Ramasamy R Yasawardena S Zomer A Venema G Kok J Leenhouts K. Immunogenicity of a malaria parasite antigen displayed by *Lactococcus lactis* in oral immunisations. Vaccine. (2006) 24:3900–8. 10.1016/j.vaccine.2006.02.040 16545511 PMC7115539

[8] van Roosmalen ML Kanninga R El Khattabi M Neef J Audouy S Bosma T. Mucosal vaccine delivery of antigens tightly bound to an adjuvant particle made from food-grade bacteria. Methods. (2006) 38:144–9. 10.1016/j.ymeth.2005.09.015 16414272

[9] Nganou-Makamdop K van Roosmalen ML Audouy SA van Gemert GJ Leenhouts K Hermsen CC. Bacterium-like particles as multi-epitope delivery platform for *Plasmodium berghei* circumsporozoite protein induce complete protection against malaria in mice. Malaria J. (2012) 11:50. 10.1186/1475-2875-11-50 22348325 PMC3337279

[10] Saluja V Amorij JP van Roosmalen ML Leenhouts K Huckriede A Hinrichs WL. Intranasal delivery of influenza subunit vaccine formulated with GEM particles as an adjuvant. AAPS J. (2010) 12:109–16. 10.1208/s12248-009-9168-2 20058113 PMC2844513

[11] Salva S Villena J Alvarez S. Immunomodulatory activity of *Lactobacillus rhamnosus* strains isolated from goat milk: impact on intestinal and respiratory infections. Int J Food Microbiol. (2010) 141:82–9. 10.1016/j.ijfoodmicro.2010.03.013 20395002

[12] Villena J Chiba E Tomosada Y Salva S Marranzino G Kitazawa H. Orally administered *Lactobacillus rhamnosus* modulates the respiratory immune response triggered by the viral pathogen-associated molecular pattern poly(I:C). BMC Immunol. (2012) 13:53. 10.1186/1471-2172-13-53 22989047 PMC3460727

[13] Tada A Zelaya H Clua P Salva S Alvarez S Kitazawa H. Immunobiotic Lactobacillus strains reduce small intestinal injury induced by intraepithelial lymphocytes after Toll-like receptor 3 activation. Inflamm Res. (2016) 65:771–83. 10.1007/s00011-016-0957-7 27279272

[14] Medina M Villena J Salva S Vintini E Langella P Alvarez S. Nasal administration of *Lactococcus lactis* improves local and systemic immune responses against *Streptococcus pneumoniae*. Microbiol Immunol. (2008) 52:399–409. 10.1111/j.1348-0421.2008.00050.x 18667039

[15] Barbieri N Villena J Herrera M Salva S Alvarez S. Nasally administered *Lactobacillus rhamnosus* accelerate the recovery of humoral immunity in B lymphocyte-deficient malnourished mice. J Nutr. (2013) 143:227–35. 10.3945/jn.112.165811 23269656

[16] Tomosada Y Chiba E Zelaya H Takahashi T Tsukida K Kitazawa H. Nasally administered *Lactobacillus rhamnosus* strains differentially modulate respiratory antiviral immune responses and induce protection against respiratory syncytial virus infection. BMC Immunol. (2013) 14:40. 10.1186/1471-2172-14-40 23947615 PMC3751766

[17] Srivastava A Gowda DV Madhunapantula SV Shinde CG Iyer M. Mucosal vaccines: a paradigm shift in the development of mucosal adjuvants and delivery vehicles. Acta Pathol Microbiol Immunol Scand. (2015) 123:275–88. 10.1111/apm.12351 25630573

[18] Miquel-Clopes A Bentley EG Stewart JP Carding SR. Mucosal vaccines and technology. Clin Exp Immunol. (2019) 196:205–14. 10.1111/cei.13285 30963541 PMC6468177

[19] Woodrow KA Bennett KM Lo DD. Mucosal vaccine design and delivery. Annu Rev Biomed Eng. (2012) 14:17–46. 10.1146/annurev-bioeng-071811-150054 22524387

[20] Villena J Vizoso-Pinto MG Kitazawa H. Intestinal innate antiviral immunity and immunobiotics: beneficial effects against rotavirus infection. Front Immunol. (2016) 7:563. 10.3389/fimmu.2016.00563 27994593 PMC5136547

[21] Lebeer S Vanderleyden J De Keersmaecker SC. Host interactions of probiotic bacterial surface molecules: comparison with commensals and pathogens. Nat Rev Microbiol. (2010) 8:171–84. 10.1038/nrmicro2297 20157338

[22] Villena J Chiba E Vizoso-Pinto MG Tomosada Y Takahashi T Ishizuka T. Immunobiotic *Lactobacillus rhamnosus* strains differentially modulate antiviral immune response in porcine intestinal epithelial and antigen presenting cells. BMC Microbiol. (2014) 14:126. 10.1186/1471-2180-14-126 24886142 PMC4035899

[23] Albarracin L Kobayashi H Iida H Sato N Nochi T Aso H. Transcriptomic analysis of the innate antiviral immune response in porcine intestinal epithelial cells: influence of immunobiotic lactobacilli. Front Immunol. (2017) 8:57. 10.3389/fimmu.2017.00057 28210256 PMC5288346

[24] Qiao X Li G Wang X Li X Liu M Li Y. Recombinant porcine rotavirus VP4 and VP4-LTB expressed in Lactobacillus casei induced mucosal and systemic antibody responses in mice. BMC Microbiol. (2009) 9:249. 10.1186/1471-2180-9-249 19958557 PMC2797526

[25] Li YJ Ma GP Li GW Qiao XY Ge JW Tang LJ. Oral vaccination with the porcine rotavirus VP4 outer capsid protein expressed by *Lactococcus lactis* induces specific antibody production. J Biomed Biotechnol. (2010) 2010:708460. 10.1155/2010/708460 20625406 PMC2896853

[26] Rodríguez-Díaz J Montava R Viana R Buesa J Pérez-Martínez G Monedero V. Oral immunization of mice with *Lactococcus lactis* expressing the rotavirus VP8 protein. Biotechnol Lett. (2011) 33:1169–75. 10.1007/s10529-011-0551-6 21302132

[27] Esteban LE Temprana CF Argüelles MH Glikmann G Castello AA. Antigenicity and immunogenicity of rotavirus VP6 protein expressed on the surface of *Lactococcus lactis*. Biomed Res Int. (2013) 2013:298598. 10.1155/2013/298598 23984337 PMC3741945

[28] Yin JY Guo CQ Wang Z Yu ML Gao S Bukhari SM. Directed chromosomal integration and expression of porcine rotavirus outer capsid protein VP4 in *Lactobacillus casei* ATCC393. Appl Microbiol Biotechnol. (2016) 100:9593–604. 10.1007/s00253-016-7779-y 27557715

[29] Temprana CF Argüelles MH Gutierrez NM Barril PA Esteban LE Silvestre D. Rotavirus VP6 protein mucosally delivered by cell wall-derived particles from *Lactococcus lactis* induces protection against infection in a murine model. PLoS ONE. (2018) 13:e0203700. 10.1371/journal.pone.0203700 30192869 PMC6128627

[30] Arce LP Raya Tonetti MF Raimondo MP Müller MF Salva S Alvarez S. Oral vaccination with hepatitis E Virus capsid protein and immunobiotic bacterium-like particles induce intestinal and systemic immunity in mice. Probiot Antimicrob Proteins. (2019). 10.1007/s12602-019-09598-7 31630331

[31] Wen K Liu F Li G Bai M Kocher J Yang X. *Lactobacillus rhamnosus* GG dosage affects the adjuvanticity and protection against rotavirus diarrhea in gnotobiotic pigs. J Pediatr Gastroenterol Nutr. (2015) 60:834–43. 10.1097/MPG.0000000000000694 25564808 PMC12967290

[32] Liu F Wen K Li G Yang X Kocher J Bui T. Dual functions of *Lactobacillus acidophilus* NCFM as protection against rotavirus diarrhea. J Pediatr Gastroenterol Nutr. (2014) 58:169–76. 10.1097/MPG.0000000000000197 24126832 PMC3908657

